# Determining Survey Satisficing of Online Longitudinal Survey Data in the Multicenter AIDS Cohort Study: A Group-Based Trajectory Analysis

**DOI:** 10.2196/publichealth.5240

**Published:** 2016-08-08

**Authors:** Junrui Di, Ying Li, M Reuel Friedman, Susheel Reddy, Pamela J Surkan, Steven Shoptaw, Michael Plankey

**Affiliations:** ^1^ Georgetown University Medical Center Department of Medicine, Division of Infectious Diseases Washington, DC United States; ^2^ University of Pittsburgh School of Public Health, Department of Infectious Diseases and Microbiology Pittsburgh, PA United States; ^3^ Northwestern University Feinberg School of Medicine, Division of Infectious Disease Chicago, IL United States; ^4^ Johns Hopkins University Bloomberg School of Public Health, Social and Behavioral Interventions Program Baltimore, MD United States; ^5^ University of California-Los Angeles David Geffen School of Medicine, Department of Family Medicine Los Angeles, CA United States

**Keywords:** ACASI, cohort studies, data collection, data quality, group-based trajectory analysis, reading speed, survey satisficing

## Abstract

**Background:**

Survey satisficing occurs when participants respond to survey questions rapidly without carefully reading or comprehending them. Studies have demonstrated the occurrence of survey satisficing, which can degrade survey quality, particularly in longitudinal studies.

**Objective:**

The aim of this study is to use a group-based trajectory analysis method to identify satisficers when similar survey questions were asked periodically in a long-standing cohort, and to examine factors associated with satisficing in the surveys having sensitive human immunodeficiency virus (HIV)-related behavioral questions.

**Methods:**

Behavioral data were collected semiannually online at all four sites of the Multicenter AIDS Cohort Study (MACS) from October 2008 through March 2013. Based on the start and end times, and the word counts per variable, response speed (word counts per second) for each participant visit was calculated. Two-step group-based trajectory analyses of the response speed across 9 study visits were performed to identify potential survey satisficing. Generalized linear models with repeated measures were used to investigate the factors associated with satisficing on HIV-related behavioral surveys.

**Results:**

Among the total 2138 male participants, the median baseline age was 51 years (interquartile range, 45-58); most of the participants were non-Hispanic white (62.72%, 1341/2138) and college graduates (46.59%, 996/2138), and half were HIV seropositive (50.00%, 1069/2138). A total of 543 men (25.40%, 543/2138) were considered potential satisficers with respect to their increased trajectory tendency of response speed. In the multivariate analysis, being 10 years older at the baseline visit increased the odds of satisficing by 44% (OR 1.44, 95% CI 1.27-1.62, *P*<.001). Compared with the non-Hispanic white participants, non-Hispanic black participants were 122% more likely to satisfice the HIV-related behavioral survey (OR 2.22, 95% CI 1.69-2.91, *P*<.001), and 99% more likely to do so for the other race/ethnicity group (OR 1.99, 95% CI 1.39-2.83, *P*<.001). Participants with a high school degree or less were 67% more likely to satisfice the survey (OR 1.67, 95% CI 1.26-2.21, *P*<.001) compared with those with a college degree. Having more than one sex partner and using more than one recreational drug reduced the odds of satisficing by 24% (OR 0.76, 95% CI 0.61-0.94, *P*=.013) and 28% (OR 0.72, 95% CI 0.55-0.93, *P*=.013), respectively. No statistically significant association of HIV serostatus with satisficing was observed.

**Conclusions:**

Using a group-based trajectory analysis method, we could identify consistent satisficing on HIV-related behavioral surveys among participants in the MACS, which was associated with being older, being non-white, and having a lower education level; however, there was no significant difference by HIV serostatus. Methods to minimize satisficing using longitudinal survey data are warranted.

## Introduction

Surveys have been widely used to collect data and provide important information for a wide variety of fields, including medical research [1]. However, the growing popularity of surveys to answer questions has led to a tendency to overlook the fact that surveys can involve many technical problems that can affect the accuracy and consistency of the data collected [2].

Satisficing is a particularly important and complicated issue for survey design that involves various psychological factors and attitude measurements. In an optimized survey administration, respondents will fully engage in four steps (1) interpreting the question; (2) searching memory for relevant information; (3) integrating information into summary judgment; and (4) reporting judgment [3-8]. The opposite of survey optimizing is referred to as “survey satisficing”, whereby a person engages in steps 2 or 3 half-heartedly or even skips steps 2 or 3 [3]. To be more specific, survey satisficing occurs when participants complete the questions rapidly without reading or comprehending them [8], or as described by Krosnick [9], it is the tendency of respondents to provide satisfactory but not optimal answers in order to reduce their effort. Garland et al [10] have shown with an interesting example that the survey responses containing both true answers and satisficed answers demonstrated divergent results. Therefore, learning about the presence and patterns of satisficing will help us design better surveys to maximize the quality of the responses received [11].

In 1991, Krosnick [9] built up a fundamental theory of satisficing, which has been widely referred. In his theory, he categorized satisficing into weak and strong satisficing, and he listed response strategies that were highly likely to cause satisficing and conditions that would foster satisficing. Depending on the format and features of the survey questions and response options, Vannette and Krosnick [12] categorized reasons for respondents’ satisficing into four responses, saying “don’t know” when they do know or could know, “acquiescence” (ie, agreeing with statements with which they might actually disagree), “response order effects”, (ie, respondents may be inclined to settle for the first plausible response option they identify when they are offered nominal or ordinal response choices), and “nondifferentiation in using rating scales” (ie, so-called straightlining when more thought might lead to different answers for different statements) [12]. In addition, Vannette provided a solution to design a survey to make it more difficult for respondents to engage in satisficing [13]. Four satisficing metrics have been used by Barge and Gehlbach [14] to demonstrate the measurable effect on survey results, which included “early termination”, “nondifferentiation”, “skipping items”, and “rushing”. An internal study performed by the technical staff at SurveyMonkey showed that a higher number of questions on a survey was highly correlated with faster speed answering each question, decreasing the validity of the data [15]. Kapelner and Chandler [16] suggested two treatments to prevent satisficing in online surveys: “timing control” (ie, featuring a disabled continue button for the duration of the waiting period) and “Kapcha” (ie, having a waiting period and attempting to attract the attention of respondents by sequentially “fading in” each word in the question’s directions, its prompt, and its answer choices). Daniel [8] found that questions toward the end of long surveys were more likely to attract a satisficing response, and one strategy to minimize this effect was to randomize question order without considering other effects of question order. Holbrook et al [17] linked response heaping (also referred to as “rounding” or “digit preference”) to satisficing.

Satisficing identification methods have been developed based on examining the occurrence of such “suspicious” response strategies highly associated with satisficing that Krosnick [9] listed, such as “don’t know”, “acquiescence”, and “straightlining” [12] under the assumption that more such behaviors indicate more satisficing and lower response quality. Oppenheimer et al [18] developed a methodological tool, termed “instructional manipulation check”, which provided an indirect measure of satisficing by measuring whether or not participants read the instructions. Garland et al [10] proposed a Bayesian inference approach that examined survey responses in quantitative form to recognize a “normal” pattern of results. Turner et al [19] recommended using response latencies to assess whether the respondents minimized their cognitive costs.

However, all of these reported satisficing theories, findings, and indicators used for satisificing identification focus on a single survey. For a longitudinal study in which surveys with similar sensitive questions are performed periodically, the reasons for respondents’ satisficing are more complicated. It is highly possible that participants in a longitudinal study become more familiar with the structure and response pattern of the survey questionnaire after several visits, and satisficing may occur due to this learning pattern compounded by participants’ lack of patience with carefully reading questions. Such a problem has been reported in a previous study [20], where many respondents complained that they were asked repeatedly to report detailed information that did not frequently change. Therefore, new measurable parameter(s) used to identify satisficing on multiple-visit surveys need to be investigated in a longitudinal study.

Literature has shown that under limited time conditions, as reading speed increases, the comprehension and recognition of important and unimportant information from a text subsequently deteriorates [21-24]. Moreover, for a survey questionnaire, it is more important to study the response speeding (ie, giving answers really quickly). Response speeding has been proven to be associated with “suspicious” response strategies, so that can be considered a parameter for satisficing identification. As pointed out by Krosnick [9], the longer an interview has been under way, the lower the respondent’s motivation to optimize and the more likely satisficing were to flourish. The “rushing” metrics Barge and Gehlbach [14] mentioned were essentially capturing faster response speed. Zhang and Conrad [25] suggested speeding was positively related to straightlining. Malhotra [26] found that speeding made respondents more likely to choose the options presented earlier regardless of the content. Conrad et al [27] observed an association between speeding and straightlining across a relatively small set of items. Wells et al [28] found that respondents who engage more in speeding were less likely to choose “other” and to elaborate on their answers. Bathelt and Bauknecht [29] compared computer-assisted personal interviewing (CAPI) and computer-assisted telephone interviewing (CATI) interviews with respect to the effect of speeding on satisficing and discovered that speeding was positively associated with satisficing in CAPI.

As pointed out by Zhang and Conrad [25], speeding in surveys is not only associated with “faster” responses, but the responses are also given faster than an appropriate speed threshold that was set low enough to capture answers that are unreasonably fast. However, this threshold is usually calculated based on a single survey questionnaire [14,25], which ignores the variation of survey questionnaires across visit times in a survey-based longitudinal study. Thus, using a universal threshold to identify speeding might not be appropriate in a longitudinal study.

During a Multicenter AIDS Cohort Study (MACS) semiannual visit, study participants are asked to complete an audio computer-assisted self-interviewing (ACASI) behavioral survey. Based on the study by Tourangeau and Yan [30], survey questions about illicit drug use and sexual behaviors are considered sensitive and tend to produce poor data quality. As an ongoing longitudinal study of 30 years targeting the cohort of homosexual men with human immunodeficiency virus (HIV) infection and with major involvement of sensitive questions about illicit drug use, sexual behaviors, and income, it is unclear whether or not satisficing has occurred with the MACS ACASI survey data over visit times, which can decrease response quality. Therefore, this current study aims to (1) plot the grouped trajectory of the MACS participants’ response speeds with visit times; (2) identify the MACS ACASI survey satisficers based on the trajectory analysis results of the longitudinal data; and (3) investigate the factors associated with satisficing in the MACS surveys having HIV-related highly sensitive questions.

## Methods

### Recruitment

The MACS is an ongoing prospective study of the natural and treated histories of HIV infection among men who have sex with men in the United States. A total of 6972 men were recruited (4954 in 1984-1985, 668 in 1987-1991, and 1350 in 2001-2003) at four centers located in Baltimore, MD/Washington, DC, Chicago, IL, Los Angeles, CA, and Pittsburgh, PA. The study design of the MACS has been described in detail previously [31,32], and only methods relevant to the current study are presented here. All MACS questionnaires are available on the MACS website [33]. The MACS study protocols were approved by the institutional review boards of each of the participating sites, their community partners, and community advisory boards, and informed consent was obtained from all participants. MACS participants return every 6 months for detailed interviews (ie, an ACASI survey), physical examinations, and collection of blood for laboratory testing and storage in a central repository.

In this study, we collected behavioral survey data via ACASI at all four MACS sites across 9 visits (ie, MACS visits 50-58) from October 1, 2008 to March 31, 2013. The survey had 7 sections including approximately 200 questions (since visit 56 from October 1, 2011, a short questionnaire with approximately 30 fewer questions was used) related to participant’s socio-demographics, illicit drug use, alcohol and cigarettes use, sexual activity, quality of life, pre-exposure or post-exposure prophylaxis, and sexual health since the previous visit.

### Measures

For each participant visit, the total survey time was calculated as the difference between the start and end times collected automatically by the computer system during the ACASI survey, and the total number of words of responded variables were counted. Some questions could be skipped logically depending on the responses to the previous question(s). For example, when a respondent answered “no” to the question “have you engaged in any sexual activities involving another person since your last visit”, the following 50 or more questions about sexual activity would be skipped. Unskipped questions could not be ignored; that is, participants had to give a response to an unskipped question before proceeding to the next one.

The primary outcome of interest was the response speed, which was calculated with the following formula:

Response Speed = N/T

where T is the total survey time (in seconds) and N is the total number of word counts of a responded variable. Note that the speed was calculated based on word count of a “variable” instead of word counts of a “question” because some survey question(s) corresponded to more than one variable.

Socio-demographics including race/ethnicity (non-Hispanic white, non-Hispanic black, or others), age at baseline visit (ie, visit 50), education level (less than a college degree, college degree, or higher than a college degree), HIV serostatus (HIV+ or HIV-), sexual activity (ie, had no sex since last visit, had one sexual partner since last visit, or had more than one sexual partner since last visit), and recreational drug use (ie, not used any illicit drug since last visit, used only one kind of illicit drug since last visit, used more than one kind of illicit drug since last visit) were studied to explore the factors associated with satisficing.

### Statistical Analysis

Group-based trajectory analyses [34] modeling the participant’s response speed across the 9 study visits were performed to define participants’ survey satisficing status. The model selection for the trajectory analysis was based on the largest negative Bayesian information criteria [35]. Based on the trajectory analysis results, a participant’s survey satisficing status (ie, satisficing or nonsatisficing) was defined. That is, the participants grouped with an increasing tendency of the response speed across the 9 visits via the trajectory analyses were considered satisficers. Univariate and multivariate generalized linear models with repeated measures were run to examine the associations of socio-demographic characteristics with survey satisficing. The iterative fitting algorithm was used for repeated measures in modeling to avoid the violation of the assumption of independence due to the multiple visits of the same participants. Statistical significance was evaluated at the .05 level, and ORs and 95% CIs were calculated. All analyses were performed using SAS version 9.3 (SAS Institute, Inc., Cary, NC, USA).

## Results

### Identification of Satisficers

There were a total of 14,722 participant visits from visits 50 to 58. After the exclusion of 14 participant visits without a start time, 1887 visits missing end times, and another 34 visits with illogical times recorded (eg, start time was 9:00 pm and the end time was 10:00 am), the study sample was composed of 12,787 participant visits contributed by 2138 MACS participants.

**Figure 1:**
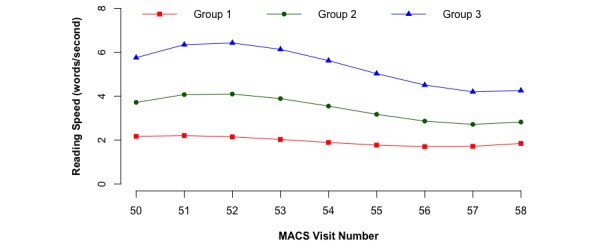
First-step trajectory analysis of response speed (word counts per second) across 9 MACS visits among the total 2138 MACS participants.

**Figure 2:**
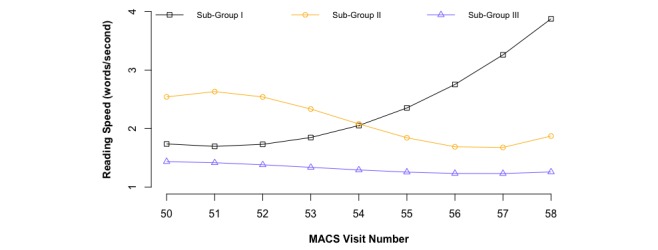
Second-step trajectory analysis of response speed (word counts per second) across 9 MACS visits among the 809 participants in group 1 of Figure 1.

The results of two-step trajectory analyses of the participants’ response speed across 9 MACS visits are plotted in Figures 1 and 2. The group-based analysis among the total of 2138 MACS participants yielded a solution that identified three groups (see Figure 1): (1) a group whose speed was approximately 6 words per second and had a decreased tendency with time (group 3; 13.14% of the cohort, 281/2138); (2) a group with a speed of approximately 4 words per second (group 2; 49.01% of the cohort, 1048/2138) showing a decreased and then constant pattern, and (3) a group whose speed of approximately 2 words per second seemed constant for the first 7 visits and then slightly increased across the recent 3 visits (group 1; 37.84% of the cohort, 809/2138). Although for group 3 response speed appeared to increase from visit 50 to visit 52, we did not consider them to be satisficing because the trajectory showed an overall decreasing pattern.

Because only group 1 showed a pattern with increased response speed during recent visits, the survey satisficers most likely existed in group 1. To observe more clear patterns, further trajectory analyses were conducted in group 1. The results of second-step trajectory analyses are plotted in Figure 2, in which three subgroups were identified. Subgroup III (32.88% of group 1, 266/809) had a nearly constant speed; subgroup I (4.33% of group 1, 35/809) revealed a dramatically increasing trajectory in response speed; and subgroup II (62.79% of group 1, 508/809) showed a decreasing pattern up to visit 56 and an accelerated speed after visit 56. Due to the accelerated response speed across the recent visits, participants in subgroups I and II were both identified as satisficers. Therefore, among the 2138 participants, 543 (25.40%, 543/2138) were identified as satisficers, and the remaining 1595 (74.60%, 1595/2138) were nonsatisficers.

### Characteristics of the Study Population by Satisficing Status

Among the total 2138 participants, the median age at baseline visit was 51 years; most were non-Hispanic white (62.72%, 1341/2138) and college graduates (46.59%, 996/2138), half were HIV seropositive (50.00%, 1069/2138), and 543 (25.40%, 543/2138) were identified as survey satisficers (Table 1). Among the 543 satisficers, 49.36% (268/543) were non-Hispanic white, 35.73% (194/543) non-Hispanic black, 54.70% (297/543) HIV seropositive, and 73.30% (398/543) had a college degree or higher. Among the 1595 nonsatisficers, 67.27% (1073/1595) were non-Hispanic white, 21.25% (339/1595) non-Hispanic black, 48.40% (772/1595) HIV seropositive, and 85.08% (1357/1595) had a college degree or higher.

**Table 1:** Characteristics of the study population by satisficing status.

		Satisficing status, n (%)		Total, n (%)
Characteristics		Nonsatisficer	Satisficer	
N		1595 (74.60)	543 (25.40)	2138 (100.00)
Baseline age in years, median (IQR^a^)		51 (45, 57)	52 (45, 59)	51 (45, 58)
Race				
	Non-Hispanic white	1073 (67.27)	268 (49.36)	1341 (62.72)
	Non-Hispanic black	339 (21.25)	194 (35.73)	533 (24.93)
	Other	183 (11.47)	81 (14.92)	264 (12.35)
Education				
	Less than college	238 (14.92)	145 (26.70)	383 (17.91)
	College	756 (47.40)	240 (44.20)	996 (46.59)
	Higher than college	601 (37.68)	158 (29.10)	759 (35.50)
HIV^b^ serostatus				
	Negative	823 (51.60)	246 (45.30)	1069 (50.00)
	Positive	772 (48.40)	297 (54.70)	1069 (50.00)

^a^IQR: interquartile range.

^b^HIV: human immunodeficiency virus.

### Factors Associated With Satisficing

In the univariate analysis, age at baseline visit, race/ethnicity, education, sexual activity, and drug use were statistically significantly associated with satisficing (Table 2). In particular, with a 10-year increase of baseline age, the odds of satisficing increased by 16% (OR 1.16, 95% CI 1.05-1.29, *P*<.001). Non-Hispanic black men and those of other race/ethnicity groups were 117% (OR 2.17, 95% CI 1.71-2.76, *P*<.001) and 60% (OR 1.60, 95% CI 1.16-2.20, *P*<.001) more likely to satisfice the survey, respectively, compared with non-Hispanic white participants. Compared with those with a college degree, the odds of satisficing were 29% lower (OR 0.71, 95% CI 0.61-0.83, *P*<.001) for participants with a high school degree or less and 15% lower (OR 0.85, 95% CI 0.74-0.98, *P*=.02) for those with a graduate degree. Compared with those without sexual activity, having one sex partner decreased the odds of satisficing by 26% (OR 0.74, 95% CI 0.61-0.91, *P*<.001), and having more than one sex partner decreased the odds by 40% (OR 0.60, 95% CI 0.49-0.73, *P*<.001). Last, the odds of satisficing were 35% lower for participants who used more than one recreational drug (OR 0.65, 95% CI 0.51-0.83, *P*<.001) than those without drug use. No statistically significant association of HIV serostatus with satisficing was observed.

**Table 2:** Odds ratios with 95% confidence intervals and *P* values of satisficing estimated with univariate and multivariate models.

		Univariate model		Multivariate model	
Socio-demographics		OR (95% CI)	*P*	OR (95% CI)	*P*
Baseline age, 10-year increase		1.16 (1.05-1.29)	.004	1.44 (1.27-1.62)	<.001
Race/ethnicity					
	Non-Hispanic white	1 (Reference)		1 (Reference)	
	Non-Hispanic black	2.17 (1.71-2.76)	<.001	2.22 (1.69-2.91)	<.001
	Other	1.60 (1.16-2.20)	.004	1.99 (1.39-2.83)	<.001
Education					
	Less than a college degree	0.71 (0.61-0.83)	<.001	1.67 (1.26-2.21)	<.001
	College degree	1 (Reference)		1 (Reference)	
	Higher than a college degree	0.85 (0.74-0.98)	.02	0.83 (0.65-1.07)	.16
HIV^a^ serostatus					
	Negative	1 (Reference)		1 (Reference)	
	Positive	1.23 (0.99-1.52)	.054	1.13 (0.90-1.42)	.29
Sexual activity					
	No sexual activity	1 (Reference)		1 (Reference)	
	Had one sex partner	0.74 (0.61-0.91)	.004	0.85 (0.69-1.05)	.14
	Had more than one partner	0.60 (0.49-0.73)	<.001	0.76 (0.61-0.94)	.013
Recreational drug use					
	No drug use	1 (Reference)		1 (Reference)	
	Used only one drug	0.92 (0.76-1.12)	.42	1.02 (0.84-1.25)	.84
	Used more than one drug	0.65 (0.51-0.83)	<.001	0.72 (0.55-0.93)	.013

^a^HIV: human immunodeficiency virus.

After considering all socio-demographic factors in a model (Table 2), a 10-year increase of baseline age increased the odds of satisficing by 44% (OR 1.44, 95% CI 1.27-1.62; *P*<.001). Compared with non-Hispanic white participants, non-Hispanic black men were 122% times more likely to satisfice the survey (OR 2.22, 95% CI 1.69-2.91, *P*<.001), and the other race/ethnicity group was 99% more likely to satisfice (OR 1.99, 95% CI 1.39-2.83, *P*<.001). Participants with a high school degree or less were 67% more likely (OR 1.67, 95% CI 1.26-2.21, *P*<.001) to satisfice the survey in comparison with those with a college degree. Having more than one sex partner and using more than one drug reduced the odds of satisficing by 24% (OR 0.76, 95% CI 0.61-0.94, *P*=.013) and 28% (OR 0.72, 95% CI 0.55-0.93, *P*=.013), respectively. We did not observe any statistically significant difference in satisficing by HIV status.

## Discussion

### Principal Findings

We studied a method using group-based trajectory analysis to investigate satisficing on the multiple-visit surveys in a longitudinal study, which would be supplementary to the traditional Krosnick’s theory focusing on a single survey. We found 25.40% (543/2138) of MACS participants were survey satisficing over time. Factors such as older age, less education, and non-Hispanic black or other race/ethnicity were positively associated with satisficing on the surveys having HIV-related sensitive questions, whereas being sexually active and using more than one recreational drug were negatively associated with satisficing. No statistically significant differences in satisficing by HIV serostatus were found.

### Comparison With Prior Work

In the fundamental Krosnick [9] theory, it was found that when question alternatives were presented to respondents visually, weak satisficing was likely to produce primacy effects. MACS used the ACASI survey, where question alternatives were visually presented to the respondent, which was potentially one source of satisficing. Moreover, in the MACS ACASI survey, when respondents were asked about quality of life and illicit drug use, the questionnaire was designed to rate a series of objects on a common scale. For example, when respondents were asked about how they feel and how things have been going during the past 4 weeks, the questionnaires just provided a list of such questions with the same scale (eg, “all of the time”, or “most of the time”). Discussed by Krosnick’s theory, this kind of questionnaire design could cause respondents to fail to differentiate between items and give all or almost all of the objects the same rating (ie, “straightlining”), and it might lead to strong satisficing [9,36].

From a survey-quality perspective, speeding has been shown to be associated with behaviors always considered indicators of survey satisficing and poor response quality [25-29]. On one hand, speeders are likely to be professional respondents solely motivated by incentives offered for survey completion and unconcerned with the survey itself or with the answers they provide. On the other hand, speeders may also be well-intentioned respondents who get frustrated with a survey (eg, too long, boring topic, contains lengthy grid items, or requires answers for every item) and react by speeding through the survey. In either case, speeding is considered problematic survey behavior because respondents are not providing thoughtful, accurate answers. One noticeable difference between speeders and non-speeders was with responses to open-ended times (questions with text boxes provided for qualitative responses), and speeders are more likely to skip these questions and not provide a response [37]. Zhang and Conrad [25] used a predefined speed threshold to detect speeding. Unfortunately, all current theories and methods about satisficing are based on a single survey, which would be not appropriate for the complicated case of a longitudinal study with several surveys on multiple visits. In this study, we used group-based trajectory analysis, a statistical approach based on mixture models that can model unobserved heterogeneity in population, to study the pattern of the trajectory of participants’ average response speed across 9 visits in a longitudinal study and to identify survey satisficing through the objective tendency of participants’ speeding across time. Moreover, our study provided abundant information regarding satisficing on such a highly sensitive survey questionnaire containing HIV-related questions for illicit drug use and sexual behaviors among men who have sex with men, those who are at high risk for HIV infection, and those who usually have poor response quality.

Data from the SurveyMonkey study demonstrated that more questions are highly associated with faster speed [15], which was in accordance with our observations at MACS after visit 56 with fewer survey questions. Less education (ie, high school degree or less) was associated with more satisficing, echoing previous studies [25-27,29,38]; as Krosnick [9] pointed out, the possible reason is that lower-status respondents were likely to agree with any assertions that the interviewer or researcher apparently believes. We found that older people were more likely to satisfice. We think it was because the elderly can become fatigued sooner, which is supported by Bathelt’s findings using the CAPI interview [29], yet differed from results of the studies by Zhang and Conrad [25] and Beckers et al [39]. Krosnick [9] stated that respondent motivation to optimize a survey (which is also a factor associated with satisficing) was influenced by the degree to which the topic of a question was personally important to the respondent. Therefore, it makes sense that participants who reported sexual activity and recreational drug use were less likely to satisfice because this population in the MACS cohort usually has higher risk for HIV infection and they may pay more attention to the survey questionnaire to better protect themselves.

### Limitations

Certain limitations of this study deserve attention. There were approximately 10% of the records missing the end time and most of these records were from one study site. Self-administration of the questionnaire did not provide an opportunity for participants to ask questions about the survey, so it was not possible to verify the accuracy of the participants’ interpretation of the survey questions, which might affect their responses. In addition, the survey did not have an independent indication of the response speed of each participant as a benchmark before they completed the questionnaire. Except for the computer system automatically recording the start and end times of an online survey, there was no other time verification method in this study.

### Conclusions

This study provides valuable insight into the identification of survey satisficing in a long-standing observational cohort study where the patterns of repetitive question asking may have been learned over time, leading to satisficing, and factors associated with satisficing on surveys having HIV-related sensitive questions. Therefore, results from the longitudinal analyses of these behavioral data could be less precise over time, even after taking into account the within-participant variability over time. Technical solutions are needed to present survey questions so that respondents read and answer questions more carefully. Survey designers should consider some basic solutions to maximize respondent motivation (eg, keep the questionnaires short and place important questions early and include random probes such as “why do you say that?”), minimize task difficulty (eg, maximize the familiarity of the words and decompose questions whenever possible), minimize response effects (eg, avoid agree/disagree, true/false, and yes/no questions, avoid blocks of ratings on the same scale, and randomize the order of list of options) [11,13,40], and/or force the participant to see the question for a certain waiting period [16].

## Abbreviations

ACASI: audio computer-assisted self interviewing
AIDS: acquired immune deficiency syndrome
CAPI: computer-assisted personal interviewing
CATI: computer-assisted telephone interviewing
CI: confidence interval
HIV: human immunodeficiency virus
IQR: interquartile range
MACS: Multicenter AIDS Cohort Study
OR: odds ratio
